# Germinated Millet (*Pennisetum glaucum* (L.) R. Br.) Flour Improved the Gut Function and Its Microbiota Composition in Rats Fed with High-Fat High-Fructose Diet

**DOI:** 10.3390/ijerph192215217

**Published:** 2022-11-18

**Authors:** Jaqueline Maciel Vieira Theodoro, Mariana Grancieri, Mariana Juste Contin Gomes, Renata Celi Lopes Toledo, Vinícius Parzanini Brilhante de São José, Hilário Cuquetto Mantovani, Carlos Wanderlei Piler Carvalho, Bárbara Pereira da Silva, Hércia Stampini Duarte Martino

**Affiliations:** 1 Department of Nutrition and Health, Federal University of Viçosa, Viçosa 36570-900, Brazil; 2 Department of Pharmacy and Nutrition, Federal University of Espírito Santo, Alegre 29500-000, Brazil; 3 Department of Microbiology, Federal University of Viçosa, Viçosa 36570-900, Brazil; 4 Embrapa Agroindústria de Alimentos, Rio de Janeiro 23020-470, Brazil

**Keywords:** beta diversity, goblet cells, intestinal health, prebiotic, intestinal permeability, whole grain

## Abstract

Germinated millet (*Pennisetum glaucum* (L.) R. Br.) is a source of phenolic compounds that has potential prebiotic action. This study aims at evaluating the action of germinated pearl millet on gut function and its microbiota composition in *Wistar* rats fed with a high-fat high-fructose (HFHF) diet. In the first stage, lasting eight weeks, the experiment consisted of two groups: AIN-93M (n = 10) and HFHF group (n = 20). In the second stage, which lasted ten weeks, the animals of the AIN-93M group (n = 10) were kept, while the HFHF group was dismembered into HFHF (HFHF diet, n = 10) and HFHF + millet (HFHF added 28.6% of germinated millet flour, n = 10) groups. After the 18th week, the urine of the animals was collected for the analysis of lactulose and mannitol intestinal permeability by urinary excretion. The histomorphometry was analyzed on the proximal colon and the fecal pH, concentration of short-chain fatty acids (SCFA), and sequencing of microbiota were performed in cecum content. The Mothur v.1.44.3 software was used for data analysis of sequencing. Alpha diversity was estimated by Chao1, Shannon, and Simpson indexes. Beta diversity was assessed by PCoA (Principal Coordinate Analysis). The functional predictive analysis was performed with PICRUSt2 software (version 2.1.2−b). Functional traits attributed to normalized OTU abundance were determined by the Kyoto Encyclopedia of Genes and Genomes (KEGG). In the results, germinated millet flour reduced *Oscillibacter genus* and Desulfobacterota phylum, while increasing the Eggerthellaceae family. Furthermore, germinated millet flour: increased beta diversity, cecum weight, and cecum/body weight ratio; improved gut histological parameters by increasing the depth and thickness of the crypt and the goblet cell count (*p* < 0.05); reduced (*p* < 0.05) the fecal pH and mannitol urinary excretion; increased (*p* < 0.05) the propionate short-chain fatty acid concentration. Thus, germinated millet has the potential to improve the composition of gut microbiota and the intestinal function of rats fed with an HFHF diet.

## 1. Introduction

Diets rich in sugar and fat are characteristic of modern society due to the use of these ingredients in industrialized foods, such as jellies, fast foods, and soft drinks. They are being increasingly consumed by the world population due to their practicality of consumption and exalted sensorial characteristics, mainly flavor [1]. However, this diet favors the progression of metabolic changes, such as insulin resistance, dyslipidemia, adiposity [2,3], and intestinal dysbiosis [4]. Furthermore, changes in the composition of the intestinal microbiota can affect the body’s homeostasis and enable barrier rupture, thus increasing permeability and allowing lipopolysaccharide (LPS) translocation [5]. This fact leads to metabolic disorders that culminate in the promotion of non-communicable chronic diseases [6].

The gut microbiota is mainly modulated by eating habits and nutritional interventions [7]. The beneficial modulation of microbiota has been considered to interfere with the regression of metabolic changes in the body. Short-chain fatty acids, mainly butyrate, acetate, and propionate–which are metabolites of the intestinal microbiota–can change the diversity of microorganisms and improve the microbiota [8]. In addition, dietary fiber and protein are associated with increased intestinal cell proliferation, which benefits intestinal health [9,10]. Therefore, some foods can improve the population of gut microorganisms, which is associated with a reduction in metabolic changes in the body and the prevention of associated diseases [8,9].

Millet (*Pennisetum glaucum*) is a whole grain abundant in proteins, dietary fiber, minerals, and bioactive compounds [11,12,13]. It is known that the germination process of the grain can increase the content of these compounds [14,15] and reduce phytochemicals, such as phytic acid [15]. In our previous studies, we demonstrated that germinated millet flour has functional activities, acting as a hypoglycemic, antioxidant, and anti-inflammatory food [16,17]. In addition, germinated millet can reduce hyperlipidemia and hepatic steatosis in animals fed a high-fat high-fructose diet [16]. Therefore, the present study aims to evaluate the effect of the consumption of germinated millet flour on intestinal function and intestinal microbiota composition in rats fed with a high-fat high-fructose diet.

## 2. Materials and Methods

### 2.1. Grain Germination

For germination, the millet (*Pennisetum glaucum* (L.) R. Br.), cultivar BRS1502, from EMBRAPA (Rio de Janeiro, Brazil) was kept at 30 ± 2 °C and 90% relative humidity for 24 h, then dried (at 50 °C for 4 h in an air circulation oven (Nova Ética, model 400/6ND, São Paulo, Brazil)), and ground in a knife mill (Brabender^®^, Duisburg, Alemanha) with a 1.0 mm stainless steel sieve to obtain the flour, as described by [16]. The resulting flour was composed of carbohydrates (64.5%), protein (15.1%), and total dietary fiber (7.6%), which consisted of insoluble dietary fiber (7.3%) and soluble dietary fiber (0.3%). Lipids (5.4%), being composed of linoleic acid (44.87 mg), oleic acid (21.70 mg), palmitic acid (17.81 mg), stearic acid (6.05 mg), and lauric acid (4.31 mg) as the main fatty acids–besides resistant starch (3.5%)–were analyzed according to AOAC [18]. The total phenolics content was 2.2 mg GAE per g [17].

### 2.2. Experimental Design, Animals, and Diets

As experimental design (Figure S1), this experiment consisted of two stages. In the first stage (induction of metabolic changes), thirty male, 45–50-day old Wistar rats (*Rattus norvegicus,* from the Central Bioterium of the Federal University of Viçosa-Brazil) were randomized, by body weight, into groups: AIN-93M (normal control group; wt 155.98 ± 17.05 g, n = 10), which received AIN-93M diet [19]; high-fat high-fructose (HFHF) (wt 158.03 ± 17.81 g, n = 20), which received a diet rich in saturated fat (31%) and fructose (20%), for 8 weeks (adapted from [20]). In the second stage (intervention), the AIN-93M group (wt 349.94 ± 30.71 g, n = 10) was maintained, and the HFHF group was dismembered into an HFHF group (wt 366.89 ± 36.90 g, n = 10, receiving HFHF diet) and HFHF + millet group (wt 370.12 ± 36.49 g, n = 10), which received the HFHF diet with 286.3 g (28.63%) of germinated millet flour added. This amount of millet replaced dietary fiber (43.6%), starch (100%), protein (36%), and oil (39%) from HFHF diet, for another 10 weeks. Throughout the experiment, the animals received water and diet ad libitum [16].

All diets were isoproteic, with same concentrations of protein (12.0%), carbohydrate (73.2, 42.5, 40.3%), lipids (4.0, 35.0, 35.0%), and energetic density (3.78, 5.33, 5.24 Kcal/g) for AIN93-M, HFHF, and HFHF + millet groups, respectively. The diets have the same amount of fiber (55.8%), however, in the AIN93-M and HFHF diets cellulose was added and in the HFHF + millet diet, 43.6% of the fiber was delivered from germinated millet flour.

Body weight and food intake were monitored once a week. One week before euthanasia, the urine of the animals was collected for the analysis of intestinal permeability. After the 18th week, the animals were anesthetized (Isoforine^®^, Cristália, Brazil) and euthanized by cardiac puncture. The proximal colon was collected, washed with PBS (phosphate buffer solution), and kept in formaldehyde (10%) at room temperature. The cecum was weighed and its content was collected and immediately stored at −80 °C. The biochemical analyses (insulin, glucose, and triglycerides), the markers of inflammation (interleukin 10 (IL-10), peroxisome proliferator-activated receptor alpha (PPARα), nuclear factor-kappa B (NFκB p65)), oxidative stress (catalase (CAT), superoxide dismutase (SOD), and malondialdehyde (MDA)), and other markers of metabolic alterations (brown adipose tissue, toll-like receptor 4 (TLR4), and phospho-protein kinase B (pAKT)) were previously performed and published [16,17].

The Ethics Committee on the Use of Animals of the Federal University of Viçosa approved the study (CEUA/UFV; process n◦ 39/2019), which was performed following Directive 86/609/EEC of 24 November 1986.

### 2.3. Intestinal Permeability

The animals were fasted for 12 h (in the last week of the experiment) and, subsequently, received by gavage 0.5 mL of a solution containing lactulose (19 mg) and mannitol (9 mg) (Sigma-Aldrich, São Paulo/SP, Brazil). After administration, the animals were placed in individual metabolic cages where they fasted for 5 h. Then, the 24-h urine was collected, measured, recorded, and stored at −80 °C [21]. Urinary lactulose and mannitol were performed by high-performance liquid chromatography (HPLC) analysis, according to de Sá et al. [22]. The concentration of lactulose and mannitol in urine was determined by a standard curve using the standard for lactulose (24–0.14 g/L) and mannitol (9–0.14 g/L) [21,23].

### 2.4. Fecal pH

The cecum content was mixed with distilled water (1:10 m:v) using vortex (20 s). Subsequently, glass electrode of pH meter (Bel Engineering, Monza, Italy) was inserted to measure the pH [24].

### 2.5. Short-Chain Fatty Acids (SCFA) Content

The concentration of SCFAs was analyzed in cecum using the methodology proposed by [25]; the quantification of SCFAs was determined by HPLC, using a Dionex Ultimate 3000 Dual detector HPLC apparatus (Dionex Corporation, Sunnyvale, CA, USA), as described by Gomes et al. [26]. Acetic, propionic, and butyric acids were used as standards in the calibration curve.

### 2.6. Colon Histomorphometry Analysis

Semi-serialized 3 μm thick histological colon fragments were obtained on an automated rotating microtome (Reichert–Jung^®^, Genossen, Germany). The fragments were stained with hematoxylin and eosin and analyzed under an Olympus BX43 light microscope (Olympus, Japan) using a 20× objective. Twenty random fields per animal were selected by crypt quality to measure crypt depth, crypt thickness, and thickness of the circular and longitudinal muscle layers. The counting of the number of goblet cells was performed manually, with the count of all cells present in the analysis field [26]. The images were processed using the ImagePro-Plus^®^ software version 4.5 (Media Cybernetics, Rockville, MD, USA) and were performed by a trained researcher.

### 2.7. DNA Extraction and Sequencing

The extraction of genomic DNA from the cecal contents was performed using the phenol/chloroform extraction protocol [27]. PCR amplicons from the V4 region of the 16S rRNA gene were generated using primers 515F (5′GTGYCAGCMGCCGCGGTAA3′) and 806R (GGACTACNVGGGTWTCTAAT3′) in addition to primers adapted for the Illumina MiSeq platform (Illumina, San Diego, CA, USA) [28,29]. Paired-end sequencing reactions were loaded into an Illumina flow cell using the Illumina MiSeq platform at the Environmental Sample Preparation and Sequencing Facility (ESPSF), Argonne National Laboratory (Lemont, IL, USA).

The sequences obtained were deposited to Sequence Read Archive (SRA) on the National Center for Biotechnology Information (NCBI) (http://www.ncbi.nlm.nih.gov/sra, accessed on 7 July 2021) under the accession number PRJNA808059.

The Mothur v.1.44.3 software was used for data analysis [30] according to Gomes et al. [26]. The Operational Taxonomic Units (OTUs) were grouped with a 97% sequence similarity cutoff. Alpha diversity was estimated using normalized data by using Chao1, Shannon, and Simpson indexes. Beta diversity among groups was assessed by Principal Coordinate Analysis (PCoA) based on the Bray–Curtis dissimilarity index [31]. The functional predictive analysis of the metagenome was performed with PICRUSt2 software [32]. Normalized OTU abundance was identified and the assigned functional traits were predicted based on reference genomes using the Kyoto Encyclopedia of Genes and Genomes (KEGG). The most abundant metabolic processes and significant fold-change differences in functional pathways between experimental groups were plotted.

### 2.8. Statistical Analysis

The animal data normality was assessed by Kolmogorov–Smirnov normality test, followed by one-way ANOVA (analysis of variance) and post hoc of Newman–Keuls. Differences between beta diversity were analyzed by the pairwise PERMANOVA test. Statistically significant *p*-values associated with microbial clades and functions were corrected for multiple comparisons by FDR correction (Benjamini–Hochberg false discovery rate). Pearson’s correlation analysis was used to assess the relationship between changes in intestinal microbiota abundance and modulations in markers of metabolic change.

The nonparametric and independent samples were submitted to Kruskal–Wallis with Dunn’s multiple comparison test. Statistical analyses were performed using SPSS software (v. 20.0) and GraphPad Prism (v. 8.0) and used *p* < 0.05 as significance level.

## 3. Results

### 3.1. Effect of Germinated Millet Flour on Food Intake, Body Weight, and Cecum Weight

**Figure 1:**
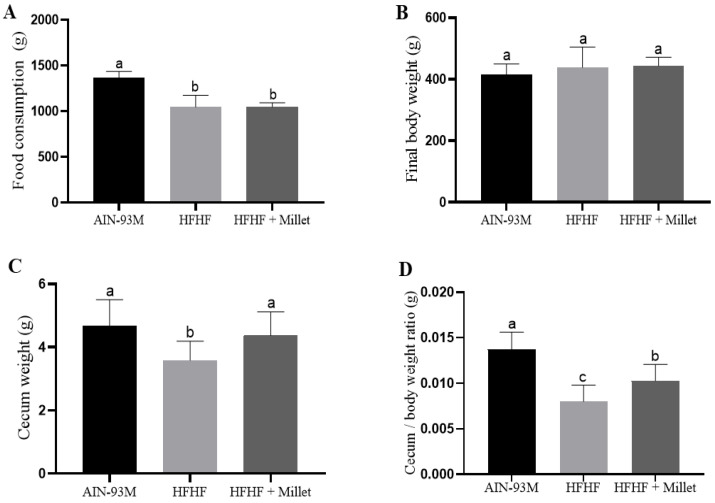
Effect of germinated millet flour on food consumption (**A**), body weight (**B**), cecum weight (**C**), and cecum/body weight ratio (**D**), after 10 weeks of intervention. Columns with different letters indicate significant differences by one-way ANOVA and post hoc of Newman–Keuls test (*p* < 0.05). Values are presented as means and standard deviation (n = 10/group). AIN-93M: standard diet; HFHF: high-fat high-fructose diet; HFHF + millet: HFHF diet + 28.63% of germinated millet flour.

After 18 weeks of the experiment (end of phase 2), the AIN-93M group had higher food intake than other groups (Figure 1A). There was no difference (*p* > 0.05) in final body weight (Figure 1B), however, the total body fat was higher (*p* < 0.05) in animals that received HFHF diet, as well as the triglycerides concentration, glucose, insulin, NFκB p65, and MDA, which characterizes these animals as metabolically obese. On the other hand, germinated millet flour increased (*p* < 0.05) the cecal weight, cecum/body weight ratio, brown adipose tissue, IL-10, PPAR-α, TLR4, SOD, CAT, and pAKT of the animals in the HFHF + millet group compared to the HFHF group (Figure 1C,D and Table S1).

### 3.2. Germinated Millet Flour Improves Intestinal Health

As for permeability, germinated millet flour reduced (*p* < 0.05) the urinary excretion of mannitol compared to the HFHF group. Urinary lactulose excretion was similar between the groups (Table 1).

**Figure 2:**
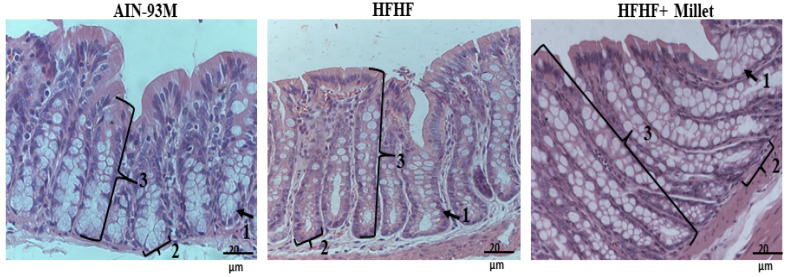
Effect of germinated millet flour on the histomorphometry of the colon. Number of goblet cells (1) and crypt thickness (2) and depth (3), after 10 weeks of intervention. AIN-93M: standard diet; HFHF: high-fat high-fructose diet; HFHF + millet: HFHF diet + 28.63% of germinated millet flour. Staining was carried out with hematoxylin and eosin. Bar: 20 μm. Objective: 20×.

Regarding other variables related to intestinal health, the group that consumed germinated millet flour (HFHF + millet flour) presented reduced fecal pH (*p* < 0.05) than the AIN-93M and HFHF groups (Table 1). As for short-chain fatty acids (SCFA), the germinated millet flour increased (*p* < 0.05) propionate production, while acetate concentration was higher in the AIN-93M group (*p* < 0.05), and butyrate concentration did not differ among the animals (Table 1). The number of goblet cells and depth of the crypts were higher (*p* < 0.05) in the HFHF + millet group than in other groups. The HFHF + millet and AIN-93M groups presented similar crypt thickness; however, this parameter was higher (*p* < 0.05) when comparing the HFHF + millet and HFHF groups (Table 1 and Figure 2). The longitudinal and circular mucus layer widths did not differ (*p* > 0.05) among the rats.

### 3.3. Effects of Germinated Millet Flour Consumption on the Diversity of the Microbial Community

To evaluate the effect of germinated millet flour consumption on intestinal microbiota, 16S rRNA gene sequencing was performed on the cecal content. This sequencing generated 765.057 raw sequences that generates 583.130 sequences with good quality after filtering and cleaning the sequences. The Good’s coverage obtained in the samples was > 99%, which indicates good sequencing coverage.

**Figure 3:**
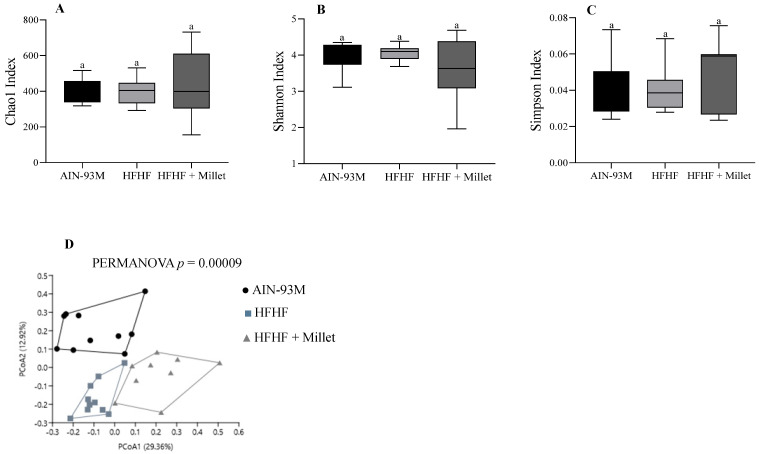
Microbial diversity of the cecal microbiome of different experimental groups after 10 weeks of the experiment. The α-diversity by: (**A**) Chao Index; (**B**) Shannon Index; (**C**) Simpson Index; (**D**) Measure of β-diversity using Bray–Curtis dissimilarity distances separated by two principal components (PCoA). Columns with different letters indicate significant differences by one-way ANOVA and post hoc of Newman–Keuls test (*p* < 0.05). The top line of the box represents the upper bound of the data, the bottom line indicates the lower bound, and the middle of the box represents the median. PCoA was analyzed by the PERMANOVA test. Each dot refers to one animal. n = 10/group (AIN-93M, HFHF groups) and n = 9/group (HFHF + millet group). AIN-93M: standard diet; HFHF: high-fat high-fructose diet; HFHF + millet: HFHF diet + 28.63% of germinated millet flour.

The alpha-diversity analysis, presented by the Chao, Shannon, and Simpson indexes, revealed no difference between the groups (*p* > 0.05) (Figure 3A–C). According to PERMANOVA, the clustering differed among all experimental groups (*p* < 0.001) (Figure 3D).

**Figure 4:**
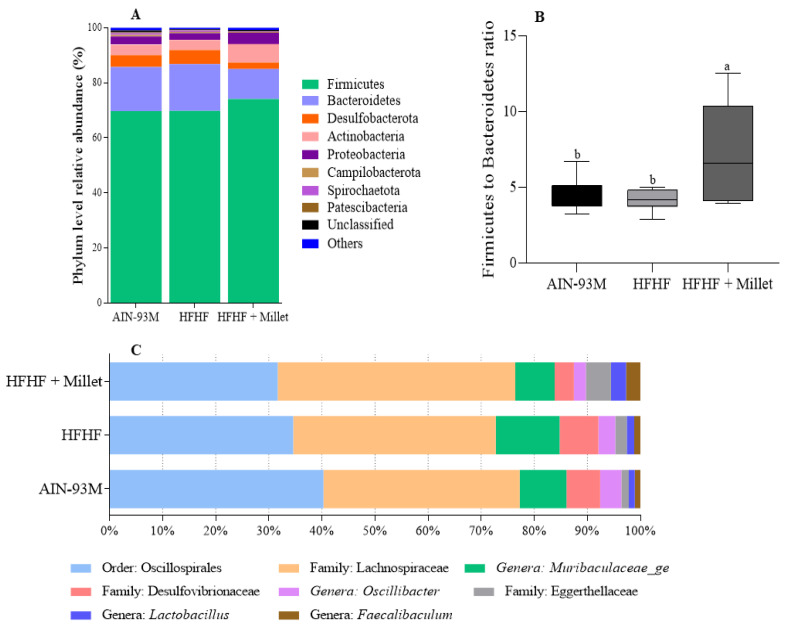
Compositional changes in intestinal microbiota in response to a germinated millet flour diet after 10 weeks. (**A**): Relative abundance of phylum; (**B**): Firmicutes/Bacteroidetes ratio; (**C**): Taxonomy level changes in each experimental group. Columns with different letters indicate significant differences by one-way ANOVA and post hoc of Newman–Keuls test (*p* < 0.05). The top line of the box represents the upper bound of the data, the bottom line indicates the lower bound, and the middle of the box represents the median. n = 10/group (AIN-93M, HFHF groups) and n = 9/group (HFHF + millet group). AIN-93M: standard diet; HFHF: high-fat high-fructose diet; HFHF + millet: HFHF diet + 28.63% of germinated millet flour. Only phyla with abundance > 0.3% in at least one group were displayed.

The taxonomic classification of the samples presented 22 phyla, 33 classes, 88 orders, 137 families, and 268 genera. Firmicutes and Bacteroidetes were the most predominant phyla in the three groups, with no difference among them (*p* > 0.05). Desulfobacterota phyla significantly decreased (*p* < 0.05) in the HFHF + millet group than AIN-93M and HFHF control groups; Actinobacteria phyla were higher (*p* < 0.05) in the HFHF + millet group than HFHF, however, similar (*p* > 0.05) to the AIN-93M control group. Further, the relative abundance of Spirochaetota and Patescibacteria phyla decreased (*p* < 0.05) in the HFHF + millet treatment group, compared to the AIN-93M control, but did not differ (*p* > 0.05) concerning the HFHF group (Figure 4A). Although the relative abundance of Firmicutes and Bacteroidetes showed no difference (*p* > 0.05), the Firmicutes to Bacteroidetes ratio was higher (*p* < 0.05) in the animals that received HFHF + millet diet than in control groups (Figure 4B).

The most abundant or key microorganisms related to SCFA production and gut health are presented in Figure 4C. The Oscillospirales order was predominant in the AIN-93M group and its relative abundance was lower in the HFHF + millet group. The relative abundance of the family Eggerthellaceae increased (*p* < 0.05) in the HFHF + millet group than AIN-93M control, while the Desulfovibrionaceae relative abundance decreased (*p* < 0.05) compared to the AIN-93M and HFHF controls. The relative abundance of the Lachnospiraceae family did not differ between treatments (*p* > 0.05). According to our data, the HFHF + millet group decreased (*p* < 0.05) in levels of the genera *Muribaculaceae_ge* and increased (*p* < 0.05) in levels of *Faecalibaculum* compared to the control groups. In addition, the treatment group with HFHF + millet reduced (*p* < 0.05) the levels of *Oscillibacter* compared to the AIN-93M control. However, no difference (*p* > 0.05) was found in the *Lactobacillus* genus between the treatment groups (Figure 4C).

**Figure 5:**
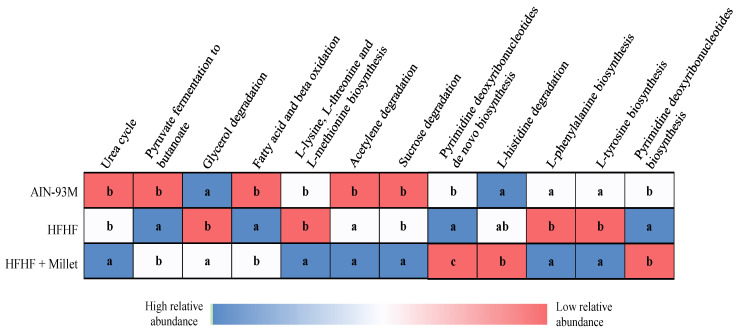
Relative significance of differentially enriched microbial metabolic pathways in cecal microbiota after the consumption of high-fat high-fructose diet associated with millet. Columns with different letters indicate significant differences by one-way ANOVA and *post hoc* of Duncan’s test (*p* < 0.05). n = 10/group (AIN-93M, HFHF groups) and n = 9/group (HFHF + millet group). AIN-93M: animals fed with a standard diet; HFHF: high-fat high-fructose diet; HFHF + millet: high-fat high-fructose diet with added germinated millet flour.

The HFHF + millet group increased (*p* < 0.05) in KEGG metabolic pathways related to the urea cycle, L-lysine, L-threonine, and L-methionine biosynthesis as well as sucrose degradation compared with controls. Furthermore, an increased relative abundance of Glycerol degradation, L-phenylalanine biosynthesis, and L-tyrosine biosynthesis pathways was observed in the HFHF + millet group compared to the HFHF group (*p* < 0.05) (Figure 5).

### 3.4. Microbial Correlation with Markers of Metabolic Alterations

When evaluating correlations with inflammatory markers, the anti-inflammatory interleukin-10 (IL-10) was positively correlated with the Eggerthellaceae family (rs = 0.520; *p* < 0.05) and negatively correlated with the Desulfobacterota phylum (rs = −0.544; *p* < 0.05) and the *Oscillibacter* genus (rs = −0.490; *p* < 0.05). The p65- NF-κB (nuclear factor Kappa B) was positively correlated with the Desulfobacterota (rs = 0.797; *p* < 0.05) and negatively correlated with the Eggertellaceae (rs = −0.711; *p* < 0.05).

The glucose homeostasis marker phospho-AKT1 [pS473] (pAKT) was negatively correlated with the Desulfobacterota (rs = −0.607; *p* < 0.05) and positively correlated with the Eggerthellaceae (rs = 0.836; *p* < 0.05). When evaluating correlations with short-chain fatty acids, acetic acid was positively correlated with the *Oscillibacter* (rs = 0.578; *p* < 0.05), and propionic acid was positively correlated with the Eggertellaceae (rs = 0.695; *p* < 0.05), IL-10 (rs = 0.781; *p* < 0.05), and pAKT (rs = 0.743; *p* < 0.05). On other hand, acetic acid was negatively correlated with the Desulfobacterota (rs = −0.480; *p* < 0.05), glucose (rs = −0.650; *p* < 0.05), and triglycerides (rs = −0.611; *p* < 0.05). The butyric acid was positively correlated with insulin (rs = 0.706; *p* < 0.05) and acetic acid (rs = 0.466; *p* < 0.05).

**Figure 6:**
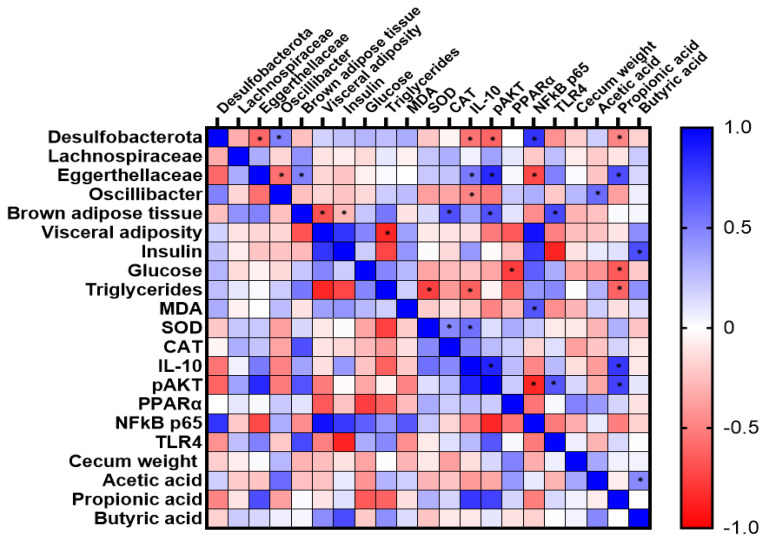
Heat map of Pearson’s correlations between markers of gut microbiota metabolism and markers of metabolic change after 10 weeks of treatment with germinated millet flour. Catalase (CAT); Interleukin 10 (IL−10); Malondialdehyde (MDA); Nuclear factor-kappa B (NFκB p65); phospho-protein kinase B (pAKT); Peroxisome proliferator-activated receptor alpha (PPARα); Superoxide dismutase (SOD); Toll-like receptor 4 (TLR4). n = 10/group. * *p* < 0.05. Data from chemical analyses, markers of inflammation and oxidative stress, and other markers of metabolic changes were previously published [16,17].

In addition, the markers of metabolic alterations and oxidative stress were correlated with each other. Brown adipose tissue was positively correlated with CAT (rs = 0.687; *p* < 0.05), pAKT (rs = 0.676; *p* < 0.05), and TLR4 (rs = 0.716; *p* < 0.05) and negatively correlated with visceral adiposity (rs = −0.677; *p* < 0.05) and insulin (rs = −0.278; *p* < 0.05). MDA was positively correlated with NFkB p65 (rs = 0.659; *p* < 0.05); SOD was positively correlated with CAT (rs = 0.465; *p* < 0.05) and IL-10 (rs = 0.558; *p* < 0.05) and negatively correlated with triglycerides (rs = −0.750; *p* < 0.05). However, these markers showed no correlation with the intestinal microbiota (Figure 6).

## 4. Discussion

**Figure 7:**
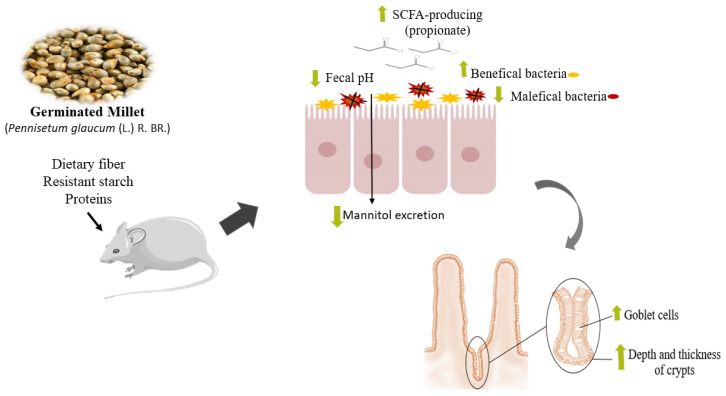
Scheme of the effects of germinated millet flour on intestinal health in rats fed with high-fat high-fructose diet. The amount of dietary fiber, proteins, and resistant starch in the germinated millet reduced the fecal pH, which improved the beta diversity providing substrate for the growth of SCFA-producing bacteria (propionate). In addition, the germinated millet flour reduced intestinal permeability, increased the depth and thickness of the crypts, and increased the number of goblet cells, improving the intestinal dysbiosis caused by HFHF diet consumption. SCFA: Short-Chain Fatty Acids.

This study evaluated the capacity of germinated millet flour to improve the intestinal dysbiosis caused by HFHF diet consumption since the germinated *Pennisetum glaucum* is rich in dietary fiber, resistant starch, and proteins. The germinated millet flour improved the characteristics of the gastrointestinal tract since it was observed, for example, with greater beta diversity, cecum weight, and cecum/body weight ratio; with increased depth and thickness of the crypts; with a greater number of goblet cells. Furthermore, the fecal pH and mannitol urinary excretion were reduced, while propionate SCFA production increased (Figure 7).

The pH was reduced by the germinated millet flour, thus improving microbial diversity and providing substrate for the growth of SCFA-producing bacteria, such as Firmicutes, Bacteroidetes, and Actinobacteria. The dietary fiber and undigested proteins present in germinated millet flour were probably fermented in the intestine and produced SCFAs, such as propionate, as observed in this research. The largest production of propionate may have stimulated the development of good bacteria, such as Actinobacteria, which increases mucus secretion and the ability to promote hypertrophy of enterocytes, consequently increasing the area of the surface of the intestine.

These results can be related to the reduction in intestinal permeability by decreasing urinary mannitol excretion in the group treated with millet, which improved the dysfunction triggered by the HFHF diet. It is demonstrated that a prolonged intake of a diet rich in saturated fat induces inflammation and impairs intestinal barrier function, thus leading to intestinal dysfunction, which is a feature of diet-induced metabolic dysregulation [33].

Furthermore, the improvement in the intestinal permeability caused by germinated millet consumption can be related to the reduction in the *Oscillibacter* genus, which are microorganisms that mediate intestinal dysfunction induced by a high-fat diet [33]. *Oscillibacter* may directly regulate the components involved in the preservation of the intestinal barrier [33]. In addition, millet consumption increased Lachnospiraceae, which has been associated with the improvement of intestinal barrier function [34].

The Desulfobacterota phylum decreased and the Eggerthellaceae family increased with the consumption of germinated millet flour. These results may be related to the high production of propionic acid, which was correlated with higher pAKT and IL-10, and a reduction in NFκB p65. This result agrees with our previous studies that used the same experimental design and demonstrated the ability of germinated millet flour to improve glucose homeostasis and reduce adipogenesis, inflammation, and oxidative stress [16,17]; however, in the present study, we did not observe a correlation between adiposity and oxidative stress markers and the microbiota. The *Lactobacillus* genus increased in the animals fed with a millet diet (HFHF + millet) but the increase was not significant. This genus has been associated with probiotic properties and demonstrated their ability to improve inflammation and intestinal barrier function in experimental models of bowel dysfunction [33].

In addition to this, the millet consumption increased the cecum weight and cecum/body weight ratio, increased the depth and thickness of the crypts, and increased the number of goblet cells, due to the higher amount of dietary fiber and resistant starch present in the germinated millet. Dietary fiber acts as a prebiotic and affects the solubility and viscosity of the feces besides stimulating or inhibiting its distension and improving the weight and motility of the intestinal [35], which can increase the thickness of muscle layers. However, there was a lack of significant changes in the thickness of the circular and longitudinal muscle layers of the colon, which agrees with Gomes et al. [35] and Moraes et al. [36]. This is possibly due to the predominance of insoluble fiber in our germinated millet flour sample [17] since the soluble fiber is mainly responsible for muscular layer increase [37]. The highest number of goblet cells, observed in the millet group, probably demonstrates a greater production of mucus in the intestinal lumen that, in turn, improves the digestion, absorption, and bioavailability of dietary components [38], possibly improving the intestinal health and systemic nutrition.

The lower food intake observed in HFHF and HFHF + millet was probably due to the high energy density provided by this diet, which reduces consumption by improvement of satiety. No change in body weight, despite increasing total body fat, was observed in HFHF group. Other studies that offered a high-fat diet for the animals also observed reduced consumption with no changes in body weight compared to the control diet (AIN-93M) [38,39,40,41].

The functional analysis of the KEGG microbial metabolic pathway observed that the HFHF + millet group positively regulated the pathways related to the urea cycle, amino acid biosynthesis (L-lysine, L-threonine, L-methionine, L-phenylalanine, and L-tyrosine) and degradation of sucrose and glycerol, which demonstrates that the germinated millet flour was able to modulate essential metabolic pathways. After amino acid metabolism, the activation of the urea cycle, which is a vital process, converts ammonia as a toxic byproduct into urea, thus allowing unwanted hydrogen to be eliminated from the body [42,43]. The degradation of sucrose and glycerol is also essential to generate energy and maintain homeostasis, in addition to amino acid biosynthesis, which still plays a key role in the immune response and oxidative stress. In our previous studies, we demonstrated that the polyphenols, dietary fiber, and resistant starch present in germinated millet flour were able to improve glucose metabolism [17] and reduce inflammation and oxidative stress [16]. These dietary components altered the microbiota allowing such modulations.

Given the composition of ungerminated millet that can be found widely described in the literature, we believe that the biological effects could also be beneficial due to the amount of dietary fiber present in the grain. However, it is demonstrated that the germinated millet has an improvement in its composition, since germination improves the bioavailability of nutrients in millet, such as proteins, vitamins, and minerals, due to the reduction in phytate content. In addition, it is demonstrated that the germination of millet reduces its naturally present compound glycosyl flavone, which can inhibit the thyroid enzyme peroxidase that is responsible for the production of thyroid hormones; therefore, germination could increase the safety of product consumption [11,44]. Thus, the germination of millet is a promising process, demonstrating good results, as observed in this study.

## 5. Conclusions

Our results demonstrate that the consumption of germinated millet flour, even associated with a high-fat high-fructose diet, improves the morphology, function, and gut microbiota composition by increasing beta diversity, Actinobacteria phyla, cecum weight, cecum/body weight ratio, depth, the thickness of the crypts, and the number of goblet cells. Furthermore, the fecal pH and mannitol urinary excretion were reduced, while propionate SCFA production increased. These results were still beneficially correlated with inflammatory and glucose homeostasis markers. Therefore, the present study highlights the potential of this flour to improve intestinal health; however, further in vivo studies are necessary to verify whether those effects are maintained in human microbiota and if different doses of millet and times of the experiment can change the observed results.

## Figures and Tables

**Table 1:** Effects of germinated millet flour intake for 10 weeks on intestinal health in *Wistar* rats (n = 8).

Variables	AIN-93M	HFHF	HFHF + Millet
Fecal pH	9.18 ± 0.26 ^a^	9.28 ± 0.16 ^a^	8.63 ± 0.38 ^b^
Propionic short-chain fatty acid (mM)	4.58 ± 1.13 ^b^	4.36 ± 1.55 ^b^	9.96 ± 3.64 ^a^
Acetic short-chain fatty acid (mM)	21.36 ± 5.11 ^a^	13.71 ± 2.90 ^b^	13.81 ± 3.05 ^b^
Butyric short-chain fatty acid (mM)	2.51 ± 0.77 ^a^	3.60 ± 1.01 ^a^	3.19 ± 0.82 ^a^
Number of goblet cells	24.64 ± 3.93 ^b^	23.88 ± 3.91 ^b^	30.57 ± 2.10 ^a^
Crypt’s depth (µM)	127.2 ± 12.06 ^b^	134.3 ± 12.21 ^b^	168.7 ± 22.34 ^a^
Crypt’s thickness (µM)	18.27 ± 1.54 ^ab^	17.16 ± 1.79 ^b^	21.12 ± 2.22 ^a^
Longitudinal mucus layer width (µM)	86.51 ± 22.88 ^a^	74.05 ± 27.48 ^a^	81.93 ± 5.81 ^a^
Circular mucus layer width (µM)	28.50 ± 7.72 ^a^	25.99 ± 8.36 ^a^	32.41 ± 8.69 ^a^
Mannitol urinary excretion (%)	2.82 ± 0.85 ^b^	9.37 ± 6.70 ^a^	4.67 ± 2.09 ^b^
Lactulose urinary excretion (%)	5.44 ± 1.37 ^a^	9.07 ± 2.99 ^a^	10.03 ± 4.91 ^a^

The line with different letters indicates significant differences by one-way ANOVA and post hoc of Newman–Keuls test (*p* < 0.05). Values are presented as means and standard deviation (n = 10/group). Short-chain fatty acids, mannitol, and lactulose were analyzed by HPLC. Histology was performed using a 20× objective and staining with hematoxylin and eosin. AIN-93M: standard diet; HFHF: high-fat high-fructose diet; HFHF + millet: HFHF diet + 28.63% of germinated millet flour.

## Data Availability

Not applicable.

## Supplementary Materials

The following supporting information can be downloaded at: https://www.mdpi.com/article/10.3390/ijerph192215217/s1, Figure S1: Experimental design. The experiment was divided into two stages: Stage 1: induction of metabolic changes: the control group received a standard rodent diet (AIN-93M); and high fat high fructose group (HFHF) received the AIN-93M diet rich in saturated fat (31%) and fructose (20%), for eight weeks. Stage 2: Intervention: control group AIN-93M was maintained and group HFHF was divided into: HFHF group and HFHF + millet group (HFHF with germinated millet flour replacing 43.6% dietary fiber, 100% starch, 36% protein and 39% oil in experimental diet) for 10 weeks; Table S1: Effect of germinated millet flour intake on biochemical variables, markers of inflammation, oxidative stress, and other metabolic alterations, in *Wistar* rats (n = 8), for 10 weeks.
