# Supplementary material for: Germinated Millet (Pennisetum glaucum (L.) R. Br.) Flour Improved the Gut Function and Its Microbiota Composition in Rats Fed with High-Fat High-Fructose Diet
Source: Int J Environ Res Public Health. 2022 Nov 18;19(22):15217. doi: 10.3390/ijerph192215217 (PMC9690063; doi:10.3390/ijerph192215217)
Supplement: Supplementary file 1 [file ijerph-19-15217-s001.zip › ijerph-1892515-supplementary.pdf]

Supplementary Material

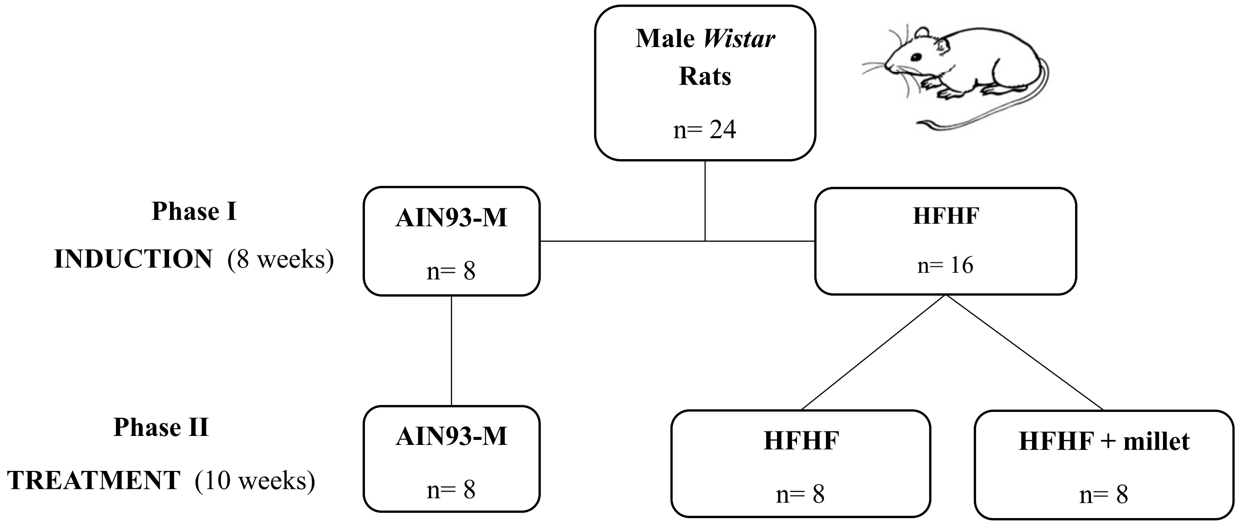

Figure S1. Experimental design. The experiment was divided into two stages: Stage 1: induction of metabolic changes: the control group received a standard rodent diet (AIN-93M); and high fat high fructose group (HFHF) received the AIN-93M diet rich in saturated fat (31%) and fructose (20%), for eight weeks. Stage 2: Intervention: control group AIN-93M was maintained and group HFHF was divided into: HFHF group and HFHF + millet group (HFHF with germinated millet flour replacing 43.6% dietary fiber, 100% starch, 36% protein and 39% oil in experimental diet) for 10 weeks.

Table S1. Effect of germinated millet flour intake on biochemical variables, markers of inflammation, oxidative stress, and other metabolic alterations, in *Wistar* rats (n = 8), for 10 weeks.

Variables	AIN-93M	HFHF	HFHF+Millet
Triglycerides (mg/dL)	117.80 ± 20.37[b]	147.80 ± 21.57[a]	100.00 ± 17.93[b]
Glucose (mg/dL)	86.00 ± 7.21[b]	100.90 ± 7.82[a]	87.50 ± 14.27[b]
Insulin (μ UI/mL)	13.74 ± 2.37[b]	34.15 ± 7.84[a]	16.49 ± 4.93[b]
NFκB-p65 (pg/mL)	226.70 ± 60.93[b]	346.30 ± 58.88[a]	131.90 ± 97.14[b]
IL-10 (pg/mL)	450.30 ± 134.40[b]	483.80 ± 91.59[b]	1511.00 ± 528.50[a]
PPAR-α (ng/mL)	3.63 ± 0.29[a]	3.05 ± 0.20[b]	3.65 ± 0.30[a]
MDA (nM/mg PTN)	351.70 ± 56.96[b]	411.10 ± 47.09[a]	343.90 ± 28.98[b]
SOD (U/mg PTN)	1.84 ± 0.29[b]	1.65 ± 0.34[b]	2.14 ± 0.25[a]
CAT (µmol/mim/mL)	25.19 ± 7.31[b]	26.13 ± 8.47[b]	35.03 ± 8.22[a]
TLR4 (ng/mL)	7.25 ± 1.14[b]	7.99 ± 1.25[b]	8.99 ± 0.67[a]
pAKT (U/mL)	1.48 ± 0.28[b]	1.76 ± 0.61[b]	3.31 ± 0.77[a]
Total body fat (g)	20.08 ± 2.67[b]	32.89 ± 8.46[a]	24.18 ± 4.45[b]
Brown adipose tissue (g)	1.14 ± 0.18[b]	1.23 ± 0.23[b]	1.47 ± 0.16[a]

Line with the different letters indicate significant differences by one-way ANOVA and *post-hoc* of Newman-Keuls test ($p<0.05$). Values are presented as means and standard deviation. NFκB p65: nuclear factor-kappa B; IL-10: interleukin 10; PPARα: peroxisome proliferator-activated receptor alpha; MDA: malondialdehyde; SOD: superoxide dismutase; CAT: catalase; TLR4: toll-like receptor 4; pAKT: phospho-protein kinase B. AIN-93M: standard diet; HFHF: high-fat high-fructose diet; HFHF + Millet: HFHF diet + 28.63% of germinated millet flour.
